# Observation of Simple Intransitive Actions: The Effect of Familiarity

**DOI:** 10.1371/journal.pone.0074485

**Published:** 2013-09-20

**Authors:** Julio Plata Bello, Cristián Modroño, Francisco Marcano, José Luis González–Mora

**Affiliations:** 1 Department of Physiology, Faculty of Medicine, University of La Laguna, San Cristóbal de La Laguna, Spain; 2 Hospital Universitario de Canarias (Department of Neurosurgery), S/C de Tenerife, Spain; 3 Servicio de Resonancia Magnética para Investigaciones Biomédicas (SRMIB), University of La Laguna, San Cristóbal de La Laguna, Spain; University of Maryland, College Park, United States of America

## Abstract

**Introduction:**

Humans are more familiar with index – thumb than with any other finger to thumb grasping. The effect of familiarity has been previously tested with complex, specialized and/or transitive movements, but not with simple intransitive ones. The aim of this study is to evaluate brain activity patterns during the observation of simple and intransitive finger movements with differing degrees of familiarity.

**Methodology:**

A functional Magnetic Resonance Imaging (fMRI) study was performed using a paradigm consisting of the observation of 4 videos showing a finger opposition task between the thumb and the other fingers (index, middle, ring and little) in a repetitive manner with a fixed frequency (1 Hz). This movement is considered as the pantomime of a precision grasping action.

**Results:**

Significant activity was identified in the bilateral Inferior Parietal Lobule and premotor regions with the selected level of significance (FDR [*False Discovery Rate*] = 0.01). The extent of the activation in both regions tended to decrease when the finger that performed the action was further from the thumb. More specifically, this effect showed a linear trend (index>middle>ring>little) in the right parietal and premotor regions.

**Conclusions:**

The observation of less familiar simple intransitive movements produces less activation of parietal and premotor areas than familiar ones. The most important implication of this study is the identification of differences in brain activity during the observation of simple intransitive movements with different degrees of familiarity.

## Introduction

Hand and finger movements are a common part of paradigms used in action observation functional Magnetic Resonance Imaging (fMRI) studies [1], [2]. The majority of them show the pattern of brain activation during the observation of complex and transitive movements (those implying an object interaction), but little is known about the activation produced by simple intransitive ones (those without an object interaction). Furthermore, simple hand movements are commonly used in clinical settings particularly since the introduction of and widespread use of fMRI methodology in brain mapping [3]. For example, brain activity provoked by simple hand actions is useful during the preoperative planning of brain tumor or malformation surgery near the motor areas [4], assessment of functional changes caused by stroke, and the collection of information about normal function recovery [5].

Execution and observation of hand and finger actions lead to an activation of parietal and premotor areas related to an action recognition system: the fronto - parietal mirror neuron system (MNS) [6], [7]. Mirror neurons were firstly discovered in the premotor cortex of macaques (area F5) [8], [9]: single cell recordings in these animals showed that the same neuron was activated not only when the macaque performed an action, but also when it observed the same action. This feature was termed “mirror property” and many human brain imaging experiments have demonstrated the presence of neural systems with mirror properties in human brain regions, anatomically comparable to the monkey’s mirror neuron areas [10]. In this sense, it is widely accepted that parietal regions (Superior Parietal Lobule [SPL], Inferior Parietal Lobule [SPL] and Intraparietal Sulcus [IPS]) and frontal regions (Dorsal and Ventral Premotor Cortex [dPMC and vPMC] and Inferior Frontal Gyrus [IFG]) are the main core of the human MNS [6].

Although the MNS of monkeys and humans share many functional and anatomical similarities, one important difference between them is the different activation produced by the observation of intransitive actions. In non-human primates, mirror neurons do not show activity during the observation of intransitive hand movements [11], but in humans there is some evidence about the activation of mirror areas during this kind of action [2], [12]–[15]. This feature allows us to use this type of movement to study the human MNS properties and compare the possible differences in the activation pattern during the observation of intransitive actions and transitive ones.

Intransitive precision grasping is a motor act which is easy and highly interesting to study. Although some brain imaging studies [16]–[19] have used this movement in their tasks (named as “thumb to index opposition task”), to the best of our knowledge no one has focused on the activity pattern related to the opposition finger task when it is performed with other fingers (thumb - middle; thumb - ring; and thumb - little).

Precision grasping is an important everyday action in human activities [20], [21]. When trying to grasp something with precision, one tends to use the thumb and index finger and the middle finger is also used depending on the features of the grasped object [20]. This movement is performed less frequently with the thumb and the middle, ring or little finger. Therefore, if familiarity in motor actions is determined by how often they are performed or observed [22], it is natural to assume that humans are more familiar with the thumb - index grasping movement than any other thumb - finger grasping movement.

The effect of familiarity has been previously tested with complex, specialized and/or transitive movements evoking more activity in parietal, frontal and cerebellar areas during the observation of familiar actions than unfamiliar ones [22]–[26].

The aim of this fMRI study is to evaluate brain activity patterns during the observation of the pantomime of an intransitive precision grasping movement performed with the right hand using the thumb and the rest of the fingers.

The hypothesis of this study is that there should be differences in brain activity for each finger grasping movement that may be the result of its different degree of familiarity. Bearing this in mind, more activity is to be expected in parietal and premotor areas during the observation of index – thumb repetitive opposition tasks than the others.

## Methods

### Subjects

Nineteen healthy, right handed (Edinburgh Handedness Inventory [27]<25) participants were selected (11 women), with an average age of 22.7 (SD = 3.1). Written informed consent was explained and signed. The study was approved by the University of La Laguna Ethics Committee, according to the Declaration of Helsinki.

### Study Design

**Figure 1:**
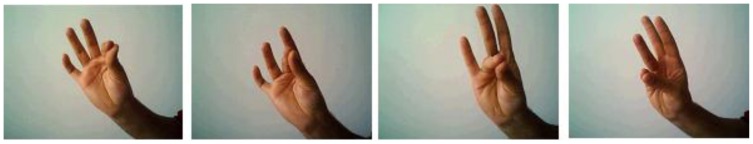
Frames of the videos which were presented to the participants. They show the performance of a precision grasp pantomime using the thumb and the rest of the fingers.

**Figure 2:**
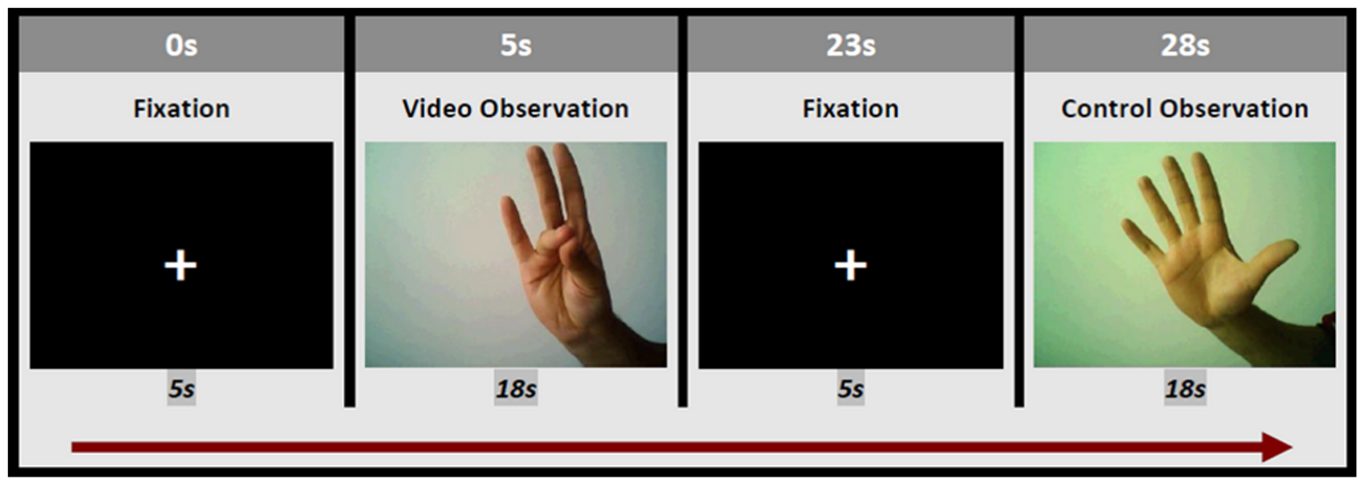
Scheme of the block design with the time onset of each condition. A total of 16 blocks were conducted (4 per finger). The upper row indicates the onset of each stage within the block. Video and control observation both lasted 18 seconds and Cross Fixation lasted 5 s.

A block design was developed in which participants observed videos which showed a right hand performing an intransitive flexion – extension movement (finger opposition task). This action can be identified as a precision grasping pantomime using the thumb and the rest of the fingers (index, middle, ring and little) (Figure 1). Four videos (one for each finger movement) were projected for 18 seconds, 4 times each. The finger movements had a frequency of 1 Hz and were presented in a third person perspective, centred on the screen. Control condition consisted of static photographs of the same hand for 18 seconds. Different grasping videos were presented in a randomized order and there was a 5 second cross fixation task (a break with participants watching a black screen with a white cross in the center of the screen) between each condition (Figure 2).

### Data Acquisition and Analysis

Data for the experiment were collected at the Magnetic Resonance for Biomedical Research Service of the University of La Laguna. Functional images were obtained on a 3T General Electric (Milwaukee, WI, USA) scanner using an echo-planar imaging gradient-echo sequence and an 8 channel head coil (TR = 1800 ms, TE = 24 ms, flip angle = 90°, matrix size = 128×128 pixels, 24 slices/volume, spacing between slices = 1 mm, slice thickness = 3 mm). The slices were aligned to the anterior commissure – posterior commissure line and covered only the part of the brain above the Silvian fissure (all parietal and frontal areas were included). Functional scanning was preceded by 18 s of dummy scans to ensure tissue steady-state magnetization.

A whole-brain three-dimensional structural image was acquired for anatomical reference. A 3D fast spoiled gradient – recalled pulse sequence was obtained with the following acquisition parameters: TR = 10.4 ms, TE = 4.2 ms, flip angle = 20, matrix size = 512×512 pixels,.5×5 mm in plane resolution, spacing between slices = 1 mm, slice thickness = 2 mm.

After checking the images for artefacts, data were preprocessed and analyzed using Statistical Parametric Mapping software SPM8 (Wellcome Trust Centre for Neuroimaging; http://www.fil.ion.ucl.ac.uk/spm/) and displayed using xjView 8.1 (http://www.alivelearn.net/xjview8/). The images were spatially realigned, unwarped, and normalized to the Montreal Neurological Institute (MNI) space using standard SPM8 procedures. The normalized images of 2×2×2 mm were smoothed by a full width at half maximum (FWHM) 8×8×8 Gaussian kernel.

A block design in the context of a general linear model was used, for individual subject analyses (first level), to look for differences in brain activity during the periods of observation and the control condition. The considered contrasts in the analysis were as follows: Index>Control (IO); Middle>Control (MO); Ring>Control (RO); and Little>Control (LO). The first-level contrast images were then used in a random-effects group analysis (second level). The group analysis was performed using an SPM8 within-subject one-way ANOVA. Violations of sphericity were allowed, in the modelling of variance components, by modelling non-independence across images from the same subject using the standard implementation in SPM8. Directional contrasts (SPM t-contrasts) were then applied to the ANOVA parameter estimates. Four tests for single regressors were conducted, one for each of the finger observation conditions. Another test was performed for the linear decrease IO>MO>RO>LO. Statistical t-maps were set at a voxel-level threshold of p<0.01, corrected with false discovery rate (FDR), and a minimum cluster size of twenty voxels. An additional F-contrast was performed to look for any difference between the four finger observation conditions (p<0.05, FDR; k = 20).

## Results

**Figure 3:**
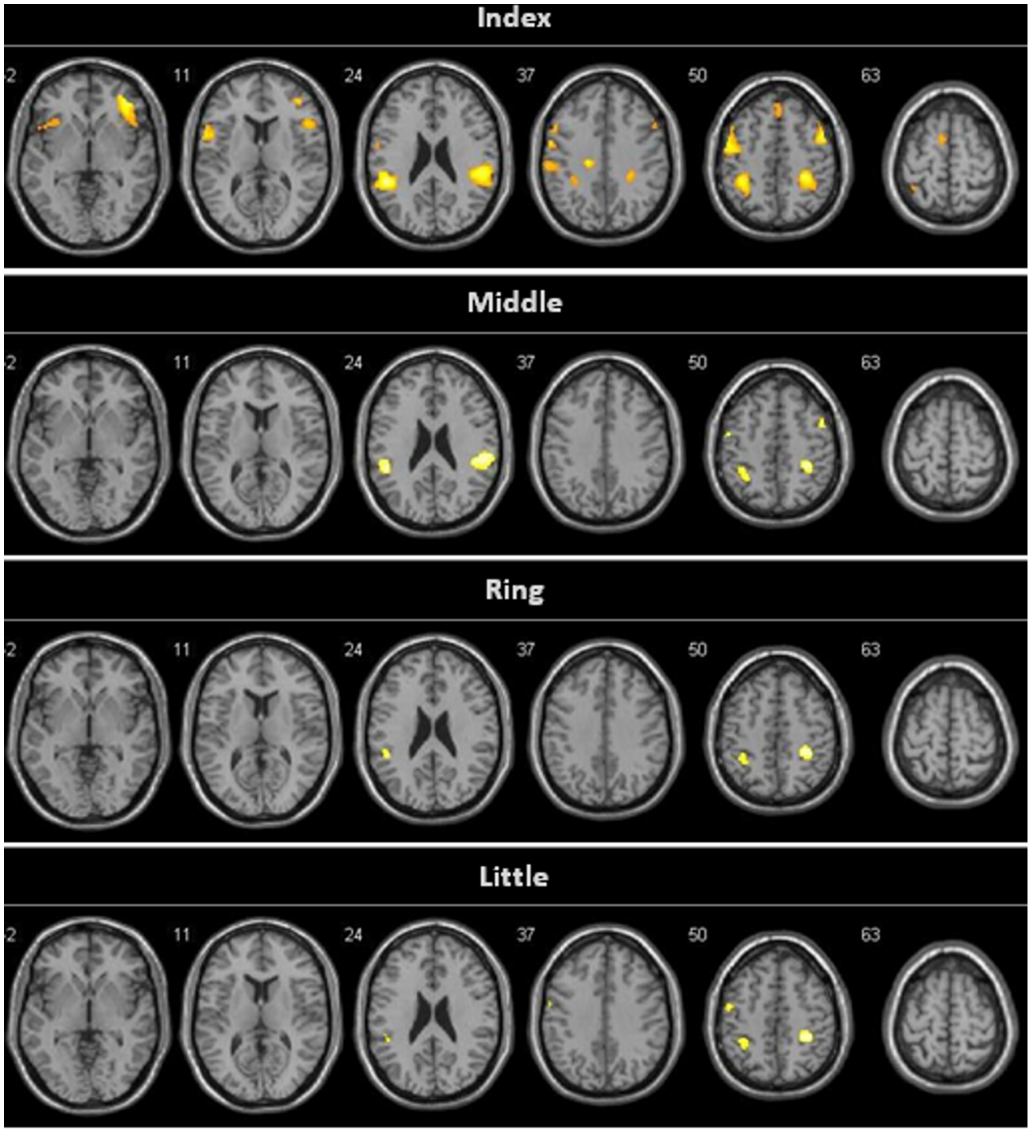
Brain activation pattern during the different finger to thumb opposition task observation. The main activation during the observation of index - thumb opposition task appears in premotor and parietal areas. As the effector finger gets further away from the thumb, the observation of the opposition task leads to a lesser activation in frontal and parietal areas. Threshold: p = 0.01, FDR; k = 20.

Significant activity appeared bilaterally in the premotor area (bilateral Superior Frontal Gyrus [SFG], right Middle Frontal Gyrus [MFG], right Inferior Frontal Gyrus and both Inferior Parietal Lobules (IPL) during the observation of index – thumb opposition task. Peaks of activation also appeared in the bilateral Postcentral Gyrus (PostCG) and left Precentral Gyrus (PreCG) (Table 1, Figure 3).

**Table 1:** Activation peaks with their locations.

Anatomical region	BA	Peak MNI coordinates				t -value	z - value	Num. voxels
		X	Y	Z				
**Index – Thumb Observation>Control**								
Right Inferior Parietal Lobule	40	52	−42	18	4.21		3.96	611
Left Inferior Parietal Lobule		−46	−40	22	6.95		6.06	617
		−34	−38	48	5.11		4.70	
		−36	−44	54	5.07		4.67	
Right Postcentral Gyrus*	2, 3	56	−30	18	6.07		5.43	219
		30	−38	48	5.44		4.96	
Left Postcentral Gyrus*		−54	−20	18	4.03		3.82	565
		−58	−24	38	4.01		3.80	
Right Inferior Frontal Gyrus	44	40	44	4	5.50		5.01	1041
		50	22	8	4.79		4.45	
Right Middle Frontal Gyrus	6	42	38	−6	5.08		4.68	441
		48	4	50	5.17		4.75	
		56	2	44	4.30		4.04	
		50	22	44	3.88		3.69	
Right Superior Frontal Gyrus		2	38	50	3.62		3.46	**53**
Left Superior Frontal Gyrus		−6	4	64	3.73		3.55	51
		−6	2	72	3.33		3.20	
Left Precentral Gyrus		−50	−4	52	5.25		4.81	555
		−58	10	8	4.82		4.47	
		−56	−2	44	4.34		4.07	
Left Cingulate Gyrus	8, 9	−16	−22	40	5.15		4.73	44
**Middle – Thumb Observation>Control**								
Right Inferior Parietal Lobule	40	56	−32	22	5,13		4,72	307
Left Inferior Parietal Lobule		−48	−40	22	5,12		4,72	237
		−34	−46	52	3,96		3,75	
Right Postcentral Gyrus	2, 3	32	−38	50	4,87		4,51	103
Right Middle Frontal Gyrus	6	50	4	48	3,99		3,78	69
Left Precentral Gyrus		−52	−6	52	3,91		3,65	20
**Ring – Thumb Observation>Control**								
Left Inferior Parietal Lobule	40	−46	−40	22	4,94		4,57	141
		−34	−46	52	4,30		4,04	
Right Postcentral Gyrus*	2, 3	30	−38	48	5,87		5,29	116
**Little – Thumb Observation>Control**								
Left Inferior Parietal Lobule	40	−32	−40	48	4,55		4,25	59
		−46	−38	22	4,52		4,22	
Right Postcentral Gyrus*	2, 3	32	−38	50	5,99		5,38	143
Left Precentral Gyrus	6	−50	−4	52	4,63		4,32	62
		−58	−2	40	4,58		4,27	
**Index>Middle>Ring>Little**								
Right Precuneus	7	10	−62	24		4,47	4,19	186
		6	−78	40		3,89	3,69	
Right Supramarginal Gyrus	40	48	−48	30		4,87	4,51	331
Right Inferior Parietal Lobule		48	−50	48		4,75	4,42	422
		64	−38	36		4,22	3,97	
Right Inferior Frontal Gyrus	44	44	30	−12		4,68	4,36	336
		38	16	−8		4,55	4,25	
Right Middle Frontal Gyrus	6	36	46	6		7,00	6,09	673
		46	12	52		4,78	4,44	
		50	22	44		4,72	4,39	
Right Superior Frontal Gyrus		28	28	54		4,99	4,61	98
		8	42	50		4,47	4,19	
		20	34	38		3,99	3,78	
Right Medial Frontal Gyrus	8, 9	10	36	36		3,80	3,62	69
Right Cingulate Gyrus		12	−44	8		4,45	4,17	309
		12	−36	26		4,34	4,08	

MNI coordinates and significance level of the respective activation cluster for *Index – Thumb, Middle – Thumb, Ring – Thumb and Little - Thumb Observation* as opposed to control condition as well as for the linear trend (Index>Middle>Ring>Little). (FDR p = 0.01; local maxima at least 8 mm apart; minimal cluster size 20 voxels). Coordinates are listed in MNI atlas space. BA is the Brodmann area nearest to the coordinate. *: These clusters extend to the Inferior Parietal Lobule of the same hemisphere.

**Figure 4:**
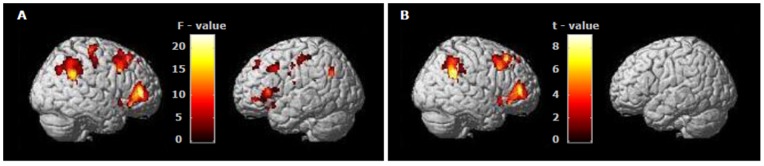
Differences in activation between the four finger observation conditions (A) and linear trend contrast (Index>Middle>Ring>Little) (B). Differences in activation between the four observation conditions appeared bilaterally in mirror areas (A) while the activation maps of the linear trend contrast show the presence of higher activity in the MNS of the right hemisphere (B). Threshold: p = 0.05 (A), p = 0.01 (B), FDR; k = 20.

The observation of the opposition task perfomed with the rest of the fingers showed peaks of activation in parietal regions of both hemispheres. The middle finger presented a bilateral IPL activation, while ring and little fingers showed left IPL activity and right parietal main activation peaks were localized in the PostCG (Table 1). However, these clusters extended to the neighboring right IPL (Figure 3). Premotor activation was found bilaterally in middle - thumb observation and only in the left hemisphere when observing little - thumb. As shown in Table 1, the extent of the clusters (number of voxels) in the parietal and premotor areas tended to decrease while the effector finger is further from the thumb. Differences in activation between the four finger observation conditions appeared bilaterally in both parietal and premotor areas (Fig. 4A). The linear trend contrast (IO>MO>RO>LO) clearly showed a linear decrease of activation in right hemisphere mirror areas (Premotor regions and IPL) as the finger used to perform the action was further away from the thumb (Table 1, Figure. 4B). The opposite contrast (IO<MO<RO<LO) did not show any significant activation in the putative MNS areas even when applying a more liberal threshold (p<0.05, FDR).

**Figure 5:**
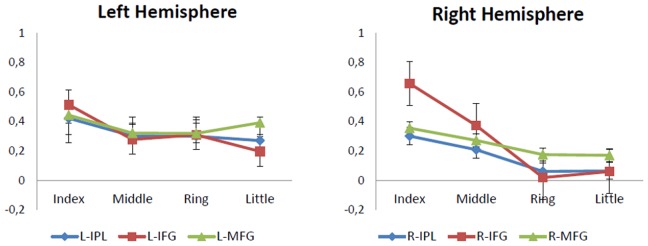
Parameter estimates in parietal and frontal regions of the left and right hemisphere. Graphics show the parameter estimates for each finger movement observation condition in six local maxima within the mirror neuron system. The error bars indicate the standard error of the mean. IPL = Inferior Parietal Lobule; IFG = Inferior Frontal Gyrus; MFG = Middle Frontal Gyrus; L/R = Left/right. MNI coordinates: R-IPL [52 −42 18]; L-IPL [−46 −40 22]; R-IFG [40 44 4]; L-IFG [−58 10 8]; R-MFG [48 4 50]; L-MFG [−50 2 52].

Figure 5 shows parameter estimates (beta values) for each of the IO, MO, RO and LO contrasts in six representative voxels. These voxels were chosen because they were points of local maxima activity during the index finger movement observation and also because they were located within in the MNS. These areas were as follows: Right Inferior Parietal Lobule [52 -42 18]; Left Inferior Parietal Lobule [-46 -40 22]; Right Inferior Frontal Gyrus [40 44 4]; Left Inferior Frontal Gyrus [-58 10 8]; Right Middle Frontal Gyrus [48 4 50]; Left Middle Frontal Gyrus [-50 2 52].

## Discussion

### Effect of Familiarity

In the present experiment, a finger dependent brain activity pattern was found during the observation of a repetitive finger opposition task which may be considered as a precision grasping pantomime. These results show the effect of familiarity in brain activity during the observation of simple and intransitive actions. It has been reported that when making a precision grasping movement, it is usually performed using the thumb and index finger and sometimes the middle finger is also used [20], [28]. In healthy subjects the use of the other fingers for this specific action is uncommon. In this sense, the index – thumb grasp may be considered as a familiar action while the others can be categorized as less familiar or unfamiliar.

The results of the present experiment show differences in the observation of the above mentioned movements and, furthermore, the activation of parietal and frontal areas is higher for familiar movements when compared to unfamiliar ones.

These results are coherent with previous reports [22]–[25], but those studies show some differential aspects with respect to the present research. They used complex, specialized and, sometimes, transitive movements. Calvo–Merino et al (2006) developed an fMRI experiment which consisted of observing videos of ballet dancers with gender specific movements. Agreeing with the present results, they described higher activity in premotor and parietal areas in both females and males when observing their most familiar action (gender specific action) [22]. Shimada (2009) also found the effect of familiarity in motor areas of baseball players who observed specific actions of not only their own position on the field but also those of a player in a different position [25].

Another difference between the above-mentioned studies and the present research is that their paradigms establish a distinction between visual and motor familiarity. The term *visual familiarity* refers to something (in this case, a motor action) that is usually seen, while *motor familiarity* is related to actions that are usually performed. A clear example was proposed by Calvo–Merino et al. (2006): “Male and female dancers train together and have equal visual familiarity with all moves” but, both have their specific movements, so they have different motor familiarity in certain motor acts [22]. We have chosen four precision grasping movements (with different degrees of familiarity). As precision grasping is a common action of great importance in everyday human activity [20], [21] and everybody is used to performing and observing it, it will be visually familiar and motorically familiar when it is performed with the index and the thumb, but it will be less familiar in both aspects when it is performed by the rest of the fingers. Therefore, a distinction between visual and motor familiarity is not applicable here.

It has been argued above that the finger to thumb opposition task can be considered as the pantomime of a precision grasping action, and Table 1 and Figure 3 show that the observation of this action leads to an activation of parietal regions in both hemispheres. Several functional studies have demonstrated similar brain activity during the execution of such actions [28]–[32]. Such a pattern of activation is why certain authors use the term “brain grasping network*”* [21], [31]. The IPL belongs to such a network [21],[31] and, as can be seen in the present study, the observation of an intransitive precision grasping pantomime leads to significant activity in that region too.

Furthermore, this activity is not only found when observing index – thumb pantomime grasping, but also when this movement is observed with the rest of the fingers. This may indicate that, when observing the finger – thumb movement, the brain understands this action as a precision grasping movement, regardless of the finger used. However, the activation of parietal regions is modulated by the finger used to perform that action, reflecting the effect of familiarity. Specifically, there is more activity in right IPL for more familiar actions, with a linear decrease as the finger gets further away from the thumb, probably because precision grasping is less common with fingers further away from the thumb.

Therefore, the experiment here shows, to the best of the authors’ knowledge, the first evidence of the effect of familiarity in brain activity during the observation of biologically simple human finger intransitive actions. This greater activity during the observation of more familiar actions may probably be due to a larger representation in the action recognition system formed by the (MNS), as is discussed below.

### MNS Activation

Activation of premotor and parietal areas during the observation of a movement is usually related to MNS activity. As described here, significant activity was found in both the IPL (bilaterally) and PMC (predominantly right) during the observation of thumb – index and bilaterally in the IPL when the effectors are the rest of the fingers. These areas are considered as an important part of the fronto–parietal mirror neuron system [6], [33]. Taking into account previous reports describing MNS activation as a result of intransitive actions [2], [12]–[15], it can be concluded that observing such grasping pantomimes also leads to MNS activation. PMC activity is shown in the results here (Table 1, Figure 3) with a local maximum in IFG, MFG (dorsal PMC) and SFG, which are also well known MNS areas [34].

Bearing in mind the effect of familiarity in action observation derived from this and other studies, it could be interpreted that MNS activity may be influenced by this factor. Motions which we are not used to performing or seeing recruit MNS less systematically, presumably because these actions do not belong to our motor repertoire [1].

Another question that emerges from the present research is why is there a finger-dependent linear decrease in the right hemisphere activity, while no linearity can be seen in the left hemisphere (Table 1; Figures 4 and 5). In short, a finger-dependent decrease in brain activity is shown for mirror areas in the left hemisphere, but it is not linear (as the linear trend contrast confirms). Differences exist, basically, between the index and the rest of the fingers, although they are not clear between the middle, ring and little finger (Figure 5A). In contrast, a finger-dependent linear decrease can be seen in the right hemisphere (Figures 4 and 5B). The results confirm the initial hypothesis in both cases, but we think the hemispherical differences in the decrease of activity in mirror areas during the observation of actions with different degrees of familiarity are also of interest.

One possible explanation for these results is that the stimuli of the present experiment consisted of a right hand and, in agreement with previous reports, greater activity can be found in the right MNS during the observation of right hand finger movements [35]. In a similar way, the degree of familiarity could have modulated the MNS mainly in the right hemisphere due to the observation of a right hand as the effector of the motor actions.

However, these differences may also be related with the fact that all the participants were right-handed, thus more familiar to grasping movements with their right hand. This could somehow balance the mirror responses for the middle, ring and little fingers in the left hemisphere, which did not happen in the right hemisphere. In any case, further experiments using left hand stimuli as well as left handed participants are necessary to clarify this point.

## Conclusions

The observation of unfamiliar simple intransitive movements produces less activation of parietal and premotor areas than familiar ones. Such differences could be related with the minor activation of the MNS for movements that are less integrated in the human motor repertoire. The most important implication of our study is the identification of differences in brain activity during the observation of simple intransitive movements with different degrees of familiarity.
